# Variceal Bleed and Portal Hypertensive Gastropathy in a Noncirrhotic Patient with Isolated Splenomegaly

**DOI:** 10.1155/2020/8893713

**Published:** 2020-12-17

**Authors:** S. M. Mahmudul Hasan, Meghan Dmitriew, Jennifer Leonard

**Affiliations:** Discipline of Medicine, Memorial University of Newfoundland, Health Sciences Centre, 300 Prince Phillip Drive, St. John's, NL A1B 3V6, Canada

## Abstract

Portal hypertension caused by cirrhosis is the most common etiology of esophageal varices. However, abnormalities of the splenoportal axis in the absence of liver disease may also cause portal hypertension resulting in varices. We report a rare case of esophageal variceal bleed in a noncirrhotic patient with isolated splenomegaly secondary to chronic granulocyte colony stimulating factor (G-CSF) therapy. The patient is a 26-year-old male with Cohen syndrome who required long-term G-CSF treatment for chronic neutropenia. He presented with large volume hematemesis and pancytopenia in the setting of known splenomegaly with no evidence of cirrhosis. An urgent EGD revealed active variceal bleeding and portal hypertensive gastropathy. The patient was appropriately resuscitated and underwent a successful transjugular intrahepatic portosystemic shunt and CT-guided coil placement for the bleeding varices. We are the first to report variceal bleed as a complication of long-term G-CSF use, a life-threatening consequence that requires urgent intervention.

## 1. Introduction

Portal hypertension is a complex clinical syndrome resulting from increased venous pressure gradient between the portal vein and the inferior vena cava. Cirrhosis is the most common cause of creating this hemodynamic alteration between the splanchnic and systemic circulation [1, 2]. Increased hepatic resistance from cirrhosis causes disruption of portal blood inflow and promotes the development of collateral vessels within the portal system leading to varices [2, 3]. However, even in the absence of cirrhosis, an increase in portal pressure due to lesions within the portosystemic axis may cause portal hypertension and its associated complications. These entities called noncirrhotic portal hypertension (NCPH) are characterized by the evidence of portal hypertension, preserved liver function, splenomegaly with or without hypersplenism, and patent hepatic and portal veins [2]. We report a rare case of a patient who presented with a variceal bleed in the context of NCPH and isolated splenomegaly secondary to chronic granulocyte colony stimulating factor (G-CSF) therapy.

## 2. Case Presentation

A 26-year-old male presented to a community hospital in rural Newfoundland with an upper gastrointestinal (GI) bleed. His past medical history was significant for Cohen syndrome, an autosomal recessive disorder involving the mutation of the vacuolar protein sorting 13 homolog B (VPS13B) gene. VPS13B is a transmembrane protein involved in intracellular protein trafficking and is critical for the development of the eye, hematological system, and central nervous system [4]. In this patient, Cohen syndrome manifested with congenital neutropenia, global developmental delay, orofacial abnormality, and retinal dystrophy.

Neutropenia in Cohen syndrome occurs from an exaggerated rate of neutrophil apoptosis and is most commonly managed with G-CSF [4, 5]. Our patient had been treated with G-CSF from the age of 13 and previous attempts to wean this had failed secondary to recurrence of severe aphthous ulcers, which affected his quality of life. His most recent bone marrow biopsy, aspiration, and cytogenetic study at age 21 were negative for malignancy and myelodysplasia.

**Figure 1:**
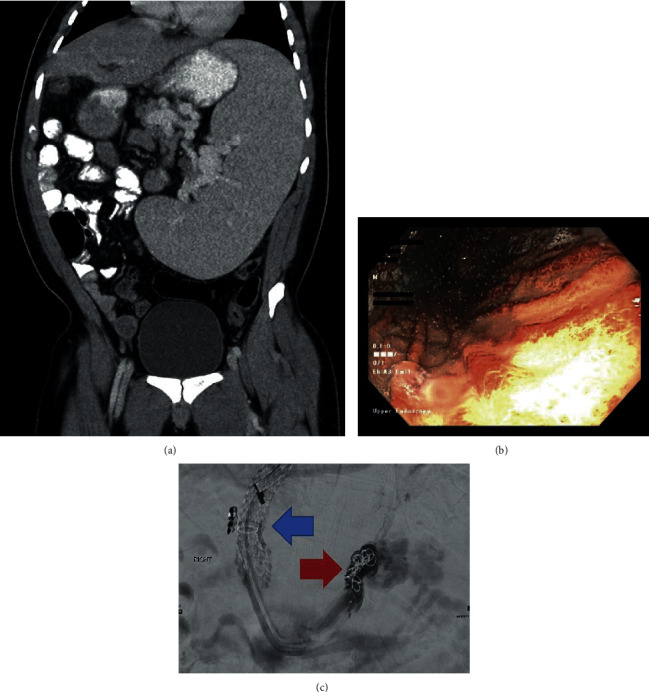
(a) Non-contrast-enhanced CT scan of the abdomen and pelvis showing splenomegaly with a craniocaudal length of 23 cm and portosystemic collaterals. (b) Endoscopic image of the stomach showing diffuse portal gastropathy. (c) Fluoroscopic image following TIPS placement (blue arrow) and coiling of the bleeding esophageal varix (red arrow).

From at least 19 years of age, our patient had worsening thrombocytopenia with a baseline platelet count between 20 and 30 × 10^9^/L. In addition to the normal bone marrow pathology, suppression of hematopoiesis from common viral infections and myeloma had been ruled out. He underwent an abdominal CT scan to rule out any structural causes of his profound thrombocytopenia and was identified to have an enlarged spleen (Figure 1(a)) at age 20. Subsequent cross-sectional imaging at age 22 was a multiplanar, multisequence abdominal MRI as per liver protocol. This illustrated stable splenomegaly with a craniocaudal length of 23 cm, normal liver size, and no radiographic evidence of cirrhosis. The imaging also identified evidence of portal hypertension such as gastroesophageal and splenorenal varices. However, there was no obvious ascites or recanalization of the umbilical vein.

Prior to his current presentation, our patient had no history of GI bleeding, frank ascites, or leg edema. His liver enzymes, bilirubin, albumin, and coagulation studies had always been within normal limits. Workup for autoimmune liver disease and viral hepatitis had been negative. The patient had no previous history of any significant alcohol intake. He had a liver biopsy completed at age 23 that was significant for mild pericellular fibrosis with no active liver disease or cirrhosis. The patient's mother was his substitute decision maker and had declined invasive venography as a mean of calculating the hepatic venous pressure gradient. A therapeutic splenectomy was also offered, but the patient and his family declined.

Our patient was doing well until he presented with sudden-onset hematemesis. He was initially taken to a community hospital where he received 4 units of packed red blood cells (pRBC) and was started on an intravenous infusion of pantoprazole and octreotide. He was then transferred to a tertiary care centre in St. John's for further resuscitation and an urgent esophagogastroduodenoscopy (EGD). On endoscopy, he had grade 3 varices which were actively bleeding, as well as diffuse portal gastropathy (Figure 1(b)). A variceal band ligator could not be passed due to the orofacial abnormalities. The patient was resuscitated with 6 more units of pRBC, 2 units of cryoprecipitate, 2 units of fresh frozen plasma, and 2 units of platelets in the Intensive Care Unit (ICU) over the next 24 hours. The following day, Interventional Radiology successfully completed a transjugular intrahepatic portosystemic shunt (TIPS) procedure with a 10 mm × 7 cm Viatorr stent along with deployment of embolization coils into the culprit varix (Figure 1(b)). This particular varix was supplied by the left gastric vein and drained through an uphill outflow into the azygous system. The patient was further monitored in the ICU and was discharged 4 days later.

## 3. Discussion

We report a rare case of variceal bleed in a noncirrhotic patient with portal hypertension from medication-induced splenomegaly. While G-CSF is thought to be generally safe for routine use in hematology and oncology patients, there are known adverse effects. Cohort studies have shown that nearly all patients receiving G-CSF develop splenomegaly, detected either by palpation or ultrasound [6]. The mechanism for this is extramedullary myelopoiesis, intrasplenic accumulation of circulating granulocytes, and trapping of stem cells. Patients receiving G-CSF are at risk of splenomegaly even if they receive it for as a little as 5 days [6–8], although this is reversible with cessation of the treatment [9]. There are multiple reports of spontaneous splenic rupture following G-CSF administration in both healthy allogenic donors undergoing apheresis and patients with chemotherapy-induced neutropenia [10–14]. Despite these well-documented complications, there is a paucity of studies assessing the long-term effects of G-CSF therapy. Only one study that followed patients for up to 11 years from the initiation of G-CSF therapy for chronic neutropenia reported a 38% increase in spleen volume over the first 5 months of treatment [15]. A literature review on PubMed and the Cochrane Library did not identify any reports associating G-CSF with variceal bleed.

An enlarged spleen causes increased flow through the splenoportal axis leading to NCPH. Splenomegaly caused by infiltrative diseases like lymphoma and myeloproliferative disease show similar phenomena [16]. Our patient had an extensive workup to rule out these possible etiologies. He also had no evidence of chronic liver disease on biopsy, and imaging of his liver revealed a normal contour with no obvious infiltration or biliary disease. Apart from G-CSF, he was not on any other regular medications. Upon reviewing medications known to cause NCPH, G-CSF is found to be the only one to mediate this effect via splenomegaly (Table 1) [2, 17, 18].

The causes of NCPH are typically classified anatomically as prehepatic, hepatic, and posthepatic. This can be differentiated based on the hepatic venous pressure gradient, which is an invasive test that helps to localize the source of resistance within the portal system [2, 3]. Our patient and his family did not want to pursue any invasive investigations. Despite not having any invasive assessments to identify the exact etiology of the patient's NCPH, by ruling out other potential causes of portal hypertension, we believe our patient to have NCPH secondary to splenomegaly.

There have been no previous studies that specifically assess the effects of portal hypertension with G-CSF treatment. However, studies in patients with NCPH have identified variceal bleeding as the most common cause of mortality and morbidity [2]. In these cases, the long-term survival after eradication of esophageal varices by endoscopic band ligation or some form of sclerotherapy was nearly 100% [19]. Nonselective beta blockers (NSBBs) are the recommended medical therapy for primary prophylaxis in patients with known small varices. In patients with medium- and large-sized varices, the recommendations are for NSBBs or endoscopic band ligation, both proving to be equally effective [19, 20]. The Baveno V Consensus suggests using the same principles to manage the risks of variceal bleeding regardless of whether the portal hypertension is caused by chronic liver disease or NCPH [2, 21].

Prophylactic surgical treatment options for varices in NCPH remain controversial. One prospective study reported that prophylactic splenorenal shunts successfully prevented variceal bleeding in patients with NCPH. However, these procedures also increase the risk of hepatic encephalopathy, glomerulonephritis, pulmonary arteriovenous fistula, and ascites [22]. As a result, patients on G-CSF will probably benefit from noninvasive imaging studies to monitor for splenomegaly and features of portal hypertension. If they do have features of portal hypertension on imaging, it is reasonable to screen for varices with an EGD, although this will largely depend on the discretion of the patient and the responsible physician [21]. In this particular case, the patient had baseline hypotension that prevented initiating any vasoactive therapy with NSBBs. The patient and his family were also not interested in pursuing an EGD or splenectomy. The combination of these challenges prevented initiating any treatment for portal hypertension.

For acute variceal bleeding, various interventional strategies including TIPS and balloon-occluded retrograde transvenous obliteration (BRTO) have been shown to be very effective [23]. In our facility, balloon-occluded retrograde transvenous obliteration (BRTO) is not commonly practiced, and TIPS is usually the standard interventional radiology procedure for variceal bleeding. Specifically, in this patient, the bleeding varix was located within the distal esophagus. While BRTO does have lower postprocedure bleeding risks and is known for improving hepatic function postprocedure, it has been primarily studied and used for gastric variceal bleeding [23, 24]. There is also some evidence that BRTO increases the portal pressure and leads to worsening of pressure within the esophageal varices [24, 25]. As a result, it was felt that TIPS was a more effective mode of intervention for reducing the overall portal pressure instead of treating the isolated bleeding varix with sclerotherapy.

There is recent conflicting evidence for G-CSF as a therapy to target the immune system and regeneration pathways in decompensated cirrhosis [26, 27]. Given its role in causing splenomegaly and associated portal hypertension, we suggest close monitoring of the portosystemic hemodynamics with the initiation of G-CSF treatment. Before committing to long-term G-CSF therapy, careful consideration must be given to specific patient attributes including preexisting portal hypertension and the use of concomitant medications that may precipitate NCPH (Table 1).

We are the first to report variceal bleed as a complication of long-term G-CSF use. The patient required an urgent TIPS procedure and variceal embolization. Complete clinical recovery ensued, and the patient was able to return home after a short stay in the ICU.

## Figures and Tables

**Table 1:** Medication and substance induced causes of noncirrhotic portal hypertension.

(i) *Prehepatic*

(a) Splenomegaly

Granulocyte colony stimulating factor (G-CSF)

(ii) *Hepatic*

(a) Presinusoidal

Vinyl chloride

(b) Sinusoidal

Methotrexate, amiodarone, vinyl chloride, copper, alcohol

(c) Postsinusoidal

Gemtuzumab ozogamicin, actinomycin D, dacarbazine, cytosine arabinoside, mithramycin, 6-thioguanine, azathioprine, busulfan, cyclophosphamide, vitamin A, alcohol, pyrrolizidine alkaloids

(iii) *Posthepatic*

Any agent causing cardiomyopathy or congestive heart failure

## Data Availability

Any further patient-specific data included in this study may be available from the corresponding author upon request. However, patient-specific information has been anonymized or omitted to maintain privacy.
